# Correction: Wang et al. Role of Berberine Thermosensitive Hydrogel in Periodontitis via PI3K/AKT Pathway In Vitro. *Int. J. Mol. Sci.* 2023, *24*, 6364

**DOI:** 10.3390/ijms25105104

**Published:** 2024-05-08

**Authors:** Chang Wang, Chang Liu, Chen Liang, Xingyuan Qu, Xinying Zou, Siyu Du, Qian Zhang, Lei Wang

**Affiliations:** 1 Department of Periodontology, Hospital of Stomatology, Jilin University, 1500 Tsinghua Road, Chaoyang District, Changchun 130021, China; wangchang20@mails.jlu.edu.cn (C.W.);; 2 Department of Prosthodontics, Hospital of Stomatology, Jilin University, Changchun 130021, China; 3 Department of Endodontics, Hospital of Stomatology, Jilin University, Changchun 130021, China

In the original publication, there was a mistake in Figure 3 as published [1]. Due to an error in the process of combining the images, the wrong image was used for the control group in Figure 3B. The corrected Figure 3 appears below. The authors state that the scientific conclusions are unaffected. This correction was approved by the Academic Editor. The original publication has also been updated.

## Figures and Tables

**Figure 3:**
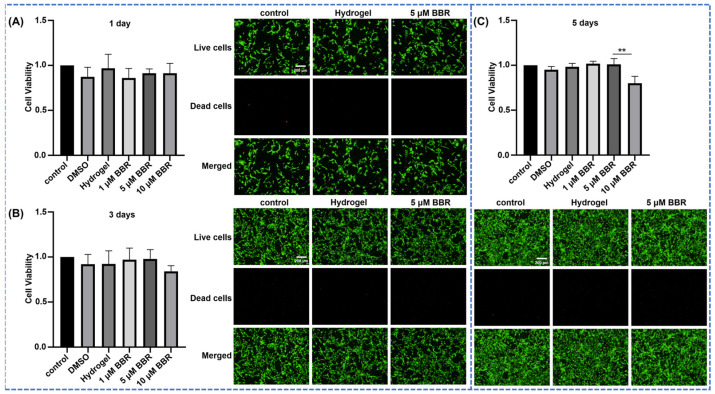
Cytotoxicity of berberine-loaded CS/β-GP/SA thermosensitive hydrogel in MC3T3-E1 cells. Viability of MC3T3-E1 cells cultured with berberine-loaded CS/β-GP/SA thermosensitive hydrogel at different concentrations (1–10 μM) at 1 Day (**A**), 3 Days (**B**) and 5 Days (**C**). Green represents living cells, and red represents dead cells. **: *p* < 0.01.
